# Epigenome-wide methylation analysis shows phosphonoethylamine alleviates aberrant DNA methylation in NASH caused by Pcyt2 deficiency

**DOI:** 10.1371/journal.pone.0320510

**Published:** 2025-03-28

**Authors:** Sophie Grapentine, Prasoon Agarwal, Vernon W. Dolinsky, Marica Bakovic

**Affiliations:** 1 Department of Human Health and Nutritional Sciences, University of Guelph, Guelph, Canada; 2 Department of Pharmacology and Therapeutics, University of Manitoba, Winnipeg, Canada; University of Alberta, CANADA

## Abstract

**Background:**

Aberrant DNA methylation can lead to the onset of pathological phenotypes and is increasingly being implicated in age-related metabolic diseases. In our preceding study we show that the heterozygous ablation of *Pcyt2,* the rate limiting enzyme in phosphatidylethanolamine (PE) synthesis, causes an age-dependent development of non-alcoholic steatohepatitis (NASH), and that treatment with the Pcyt2 substrate phosphonoethylamine (PEA) can attenuate phenotypic NASH pathologies. Here, we hypothesize that abnormal DNA methylation patterns underly the development of *Pcyt2*^* + /-*^ NASH. In this study, we conduct an epigenome-wide methylation analysis to characterize the differential methylation of *Pcyt2*^* + /-*^ livers and investigate whether the attenuation of NASH with PEA treatment is associated with changes in DNA methylation.

**Results:**

*Pcyt2*^* + /-*^ NASH liver experiences significant alterations in DNA methylation pattens relative to *Pcyt2*^* + / + *^. Differentially methylated genes belong to pathways including PI3K-Akt signalling pathway, Foxo signalling pathway, oxidative phosphorylation and insulin signalling/secretion, indicating that epigenetic regulation underlies many of our previously established functional pathological mechanisms of Pcyt*2*^* + /-*^ NASH. Previously unidentified pathways during Pcyt2 deficiency are highlighted, such as cell cycle regulation and cellular senescence that may contribute to NASH development. Treatment with PEA dramatically attenuates aberrant total and protein-coding DNA methylation patterns by 96%. PEA treatment restored the methylation status of key genes involved in epigenetic modifications and induced differential methylation of genes associated with obesity and T2DM such as *Adyc3, Celsr2, Fam63b.*

**Conclusion:**

The *Pcyt2*^* + /-*^ liver methylome and transcriptome is altered and likely underlies much of the pathology in *Pcyt2*^* + /-*^ NASH phenotype. The treatment with PEA significantly attenuates aberrant DNA methylation in *Pcyt2*^* + /-*^ liver and corrects the DNA methylation of genes involved in the pathogenesis of NASH, indicating its therapeutic potential. This analysis provides critical insight into the epigenetic basis of NASH pathophysiology and suggests diagnostic markers and therapeutic targets.

## Introduction

Non-alcoholic steatohepatitis (NASH) is a progressive and dangerous form of non-alcoholic fatty liver disease (NAFLD) characterized by hepatic steatosis with cellular injury, inflammation, and fibrosis, that can advance to cirrhosis, hepatocellular carcinoma, and death. The onset and rate of NASH progression is tightly linked to metabolic syndrome, obesity, type 2 diabetes (T2DM), and age [1]. Total prevalence of NAFLD is estimated at 28.5%, with NASH-specific cases predicted to increase 63% and liver-related deaths increase 178% from 2015 to 2030 [2].

NASH is a complex and multifaceted disease state in which the pathogenesis is incompletely understood. Currently, intervention strategies are limited to lifestyle modifications as there are no approved pharmacotherapeutics for NASH treatment [3]. Because both NAFLD and NASH are asymptomatic until late stage, patient diagnosis often occurs at a phase that is too advanced for effective lifestyle interventions. Thus, with an aging population and increasingly high rates of obesity and T2DM there is an urgent need for a deeper mechanistic understanding of NASH development to identify prognostic and diagnostic markers and facilitate pharmacological therapies to prevent disease progression.

Accumulating evidence associates epigenetic mechanisms and NASH pathogenesis. The most well-studied epigenetic modification is DNA methylation, which involves the addition of methyl groups to the 5’ position on cytosine nucleotides in CpG sequences [4]. Aberrant DNA methylation can lead to the onset of pathological phenotypes and is increasingly being implicated in age-related and metabolic diseases including NASH [5–10]. Alterations in DNA methylation can both underly disease etiology and be a consequence of the diseased state. Therefore, identifying DNA methylation changes during NASH development will help to clarify disease pathophysiology and indicate novel therapeutic targets.

Perturbed phospholipid homeostasis is emerging as an important pathological mechanism in metabolic disorders [11], and specifically, disruptions in phosphatidylethanolamine (PE) metabolism are repeatedly linked to obesity, diabetes, and fatty liver diseases [11–13]. PE is the most abundant phospholipid on the inner leaflet of cellular membranes where it is involved with essential processes such as cell signaling, membrane fission and fusion, autophagy, and the regulation of glucose, lipid, and systemic energy metabolism [12]. PE metabolism has the potential to influence DNA methylation by modulating the cellular levels of the universal methyl donor S-adenosylmethionine (SAM) as PE is a major consumer of liver SAM during the methylation of PE to phosphatidylcholine (PC) [14]. Thus, changes in PE production and turnover can influence SAM usage and availability for DNA methylation reactions.

PE is mainly synthesized *de novo* through the CDP-ethanolamine pathway. We previously generated a mouse model with the heterozygous knock out of CTP:phosphoethanolamine cytidylyltransferase (Pcyt2), the rate-limiting enzyme in the CDP-ethanolamine pathway. *Pcyt2*^* + /-*^ mice develop adult onset metabolic syndrome and NASH [15–17]. *Pcyt2*^* + /-*^ mice are an ideal model for studying NASH pathogenesis because they exhibit both the histological and inflammatory features of NASH, along with the associated metabolic physiology [15,16]. This contrasts with previous models that induce NASH using choline/methionine deficient and high-fat diets which fail to fully recapitulate NASH etiology and pathogenic changes [18–20]. *Pcyt2*^* + /-*^ mice develop NASH on a normal diet, allowing for the distinction of mechanisms underlying NASH progression from co-occurring factors associated with artificial diets used in alternative models. The *Pcyt2*^* + /-*^ mouse model is further relevant to humans because in humans, NASH and a decrease in PE synthesis associate with age [21–23] and similarly, *Pcyt2*^* + /-*^ mice do not clinically manifest symptoms at a young age, but progress to a pathological state as adults.

In our preceding study, we found that *Pcyt2*^* + /-*^ mice show changes in hepatic metabolic regulators from young age (2-mo), prior to the development of liver disease. Adult (6-8-mo) *Pcyt2*^* + /-*^ mice exhibit insulin resistance and develop NASH that is characterized by increased glucose production, accumulation of TAG and glycogen, and increased inflammation [16]. We also showed that supplementation with the artificial Pcyt2 substrate phosphonoethylamine (PEA) reverses *Pcyt2*^* + /-*^ steatosis, inflammation, and improved the dysregulation of various regulators associated with NASH pathogenesis [16]. In this study, we build on these phenotypic findings by probing the changes in hepatic methylation that associate with the development of *Pcyt2*^* + /-*^ NASH. We performed an epigenome-wide analysis of livers from *Pcyt2*^* + /-*^ NASH mice without and with PEA treatment to characterize how changes in DNA methylation may have influenced the reversal of *Pcyt2*^* + /-*^ NASH in PEA treated livers. Here we show that the *Pcyt2*^* + /-*^ NASH liver is heavily differentially methylated, and that PEA exhibits a dramatic reversal of the aberrant methylation underlying the phenotypic improvements in *Pcyt2*^* + /-*^ NASH. We further complement our findings by showing that molecular pathways that are implicated in NASH pathogenesis contain the greatest changes in DNA methylation.

## Methods

### Animals and treatments

Heterozygous Pcyt2 mice (*Pcyt2*^* + /-*^) of a mixed genetic background (C57BL/6 ×  129/Sv) were generated and genotyped as previously described (n = 3-4) [24]. Mice were housed in a temperature-controlled facility and exposed to a 12 h light/12 h dark cycle beginning with light at 7:00 a.m. Mice were fed a standardized chow diet (Harlan Teklad S-2335) and had free access to water. Mice that were supplemented with phosphonoethylamine (PEA) (PEA, Sigma-Aldrich 268674) were done so through free access to water containing 1 mg/mL of PEA. Dosage of PEA was calculated based on physiological levels of ethanolamine (10–75 μM) [25] and previously determined water intake [26]. The treatment period started at 5-6 months of age and lasted 8 weeks until sacrifice at 7-8 months. Mice were euthanized using CO2 as per approved protocols to minimize distress. Efforts to alleviate suffering included careful monitoring of animal for distress and ensure all procedures were approved by the University of Guelph’s Animal Care Committee and were conducted by trained personnel in accordance with the guidelines of the Canadian Council on Animal Care (CCAC) and the ARRIVE guidelines for reporting results. We previously observed no differences in the NASH and treatment groups were observed between age-matched males and females [16]; therefore, wild-type *Pcyt2*^* + / + *^, untreated *Pcyt2*^* + /-*^ and PEA-treated *Pcyt2*^* + /-*^ (*Pcyt2*^* + /-*^ + PEA) (n = 3-4 each) females were used for final methylation analysis.

### Preparation of methyl binding domain-biotin/streptavidin magnetic beads

Streptavidin coupled Dynabeads M-280 (Thermo Fisher Scientific) were suspended in 1X ELISA Tris-based wash buffer, washed twice on a magnetic rack, and resuspended in 100 uL of the same buffer. Methyl binding domain (MBD)-biotin protein (7 mL MBD-biotin protein +  93 mL 1X wash buffer) was added to the beads and the mixture was incubated with rotation for 1 h at room temperature. The mixture was then briefly spun, washed, and resuspended in 1X ELISA/Tris wash buffer. The biotin attached beads are additionally rotated for 5 min, washed, and resuspended in 100 uL of 1X ELISA/Tris buffer.

### Preparation of methylated genomic DNA

Genomic DNA was extracted from 20-30 mg of the whole liver homogenate frozen liver using gSYNC DNA Extraction Kit (FroggaBIO; # GS100). The methylated genomic DNA (Me-DNA) was prepared using Methyl Miner Protocol (Invitrogen -ME10025) for the 5500 series ASOLID systems platform. In brief, 5 ug of total DNA was sheared using S220 Focused-ultrasonicator (Covaris) in TruShear buffer (Covarius). Me-DNA was then captured by incubation with the MDB-biotin/streptavidin beads at room temperature for 1 h. The beads bound Me-DNA was released from the beads using HIS-Select elution buffer (Sigma-Aldrich) by 3 min rotation at room temperature. The elution step was repeated twice, and the eluted Me-DNA was ethanol-precipitated and stored at -20^o^C.

### Me-DNA-seq, data filtering and bioinformatics

The adaptor ligated Me-DNA library for the next-generation sequencing was prepared using Neb next Ultra II DNA Library Prep Kit (Illumina, #E7645). The sequencing was performed on HiSeq 2000 instrument (Illumina) using standard protocols. Dataset quality control for poor base call and adapter contamination was performed using Trim Galore tool (https://github.com/FelixKrueger/TrimGalore.git). The files were aligned using Burrows-Wheeler Aligner and short read alignment performed with Burrows-Wheeler Transform Bioinformatics default parameters [27]. The mouse genome assembly mm10 was used for aligning the reads. Picard was used to mark duplicate reads (http://broadinstitute.github.io/picard/). Differentially methylated regions (DMRs) were separated using diffReps with default parameters and the fragment size of 300 bp [28]. The input bam files were converted to bed using Bedtools bamTobed, to test for correlations between different sequences. The significant DMRs were selected with a cut-off of FDR ≤  0.05.

### Microarray and gene-expression RT-PCR arrays

mRNA from 8-month *Pcyt2*^* + / +*^ and *Pcyt2*^* + / −*^ NASH liver (n =  3) was extracted using the RNeasy Mini Kit (QIAGEN). For complementary DNA (cDNA) synthesis, 1 μg total RNA was reverse transcribed using the High-Capacity RNA to DNA Master Mix (Applied Biosystems). The gene-expression PCR arrays were carried out using an Applied Biosystems 7900HT Fast Real-time PCR system (Applied Biosystems). Insulin Signaling Pathway (Mouse Gene Expression Array PAMM-030Z) and JAK/STAT Signaling Pathway (Mouse Gene Expression Array PAMM-039Z) PCR Arrays (SA-Biosciences) were used for expression analysis of 84 genes. β-Gusβ, Hprt, Hsp90ab1, GAPDH, and Actβ were used as positive controls. PCR array data were calculated by the comparative cycle threshold method, normalized against multiple housekeeping genes, and expressed as mean fold change.

We have previously performed a microarray mRNA analysis on the liver tissue of 2-month old *Pcyt2*^* + /-*^ mice and age matched *Pcyt2*^* + / +*^ controls (n = 6) using Affymetrix Mouse Gene 1.1 ST Array (GSE55617) [29]. The overlapping genes between the microarrays and Me-DNA-seq genes were identified using Bedtools intersect with default parameters (https://bedtools.readthedocs.io/en/latest/index.html).

### Gene expression analysis

Frozen liver tissue weighing between 50-100 mg (n = 3-4 per group), was homogenized in 1 mL of TRIzol Reagent (Thermo Scientific) according to the manufacturer’s protocol to isolate mRNA. cDNA was synthesized from 2 μg of total mRNA using a poly(dT) primer and Superscript III reverse transcriptase (Invitrogen). Expression of various genes were determined by polymerase chain reaction (PCR). PCR reactions were carried out using the following cycle parameters: 30 s at 94°C, 30 s 30 s at 72°C for 32 cycles. PCR products were resolved on a 1.5% agarose gel and quantified using ImageJ. Reactions were standardized to glyceraldehyde 3-phosphate dehydrogenase (*Gapdh*).

### Functional pathways analysis

The online bioinformatic tool Enrichr (https://maayanlab.cloud/Enrichr/) [30–32] was used for the enrichment analysis of the Me-DNA-seq, RT-PCR array and microarray data. Differentially methylated and/or expressed genes were mapped to Kyoto Encyclopedia of Genes and Genomes (KEGG), BioPlanet and Gene Ontology (GO) Biological Process databases. Benjamini-Hochberg adjusted *p* value ( < 0.05) was used as the significance cut-off and represented using a z-score permutation background correction on Fisher’s exact test *p* value to generate an overall score for each pathway. This method of pathway analysis has been shown to recover the most correct terms [31]. The score plots use to display the enrichment analysis were produced using GraphPad Prism v9 software.

### Network analysis

Protein-protein interaction (PPI) networks were generated using NetworkAnalyst 3.0 [33] which uses the Search Tool for the Retrieval of Interacting Genes (STRING) database v11.5 [34]. The networks were generated using generic PPI and STRING interactome with a high confidence score cut-off (900) and requiring experimental evidence. Isolated nodes were not included in networks. Enrichment analysis of genes in networks was conducted using KEGG database. The analysis was performed on the set of genes that are both differentially methylated and expressed using the Me-DNA-seq, microarray, and RT-PCR array data.

### Statistical analysis

Data was analyzed as described in previous sections, for all other analysis GraphPad Prism v9 software was used. Principal component analysis, correlation analysis, and linear regression was used to probe the relationship between the Me-DNA-seq and microarray data. *P* values <  0.05 were considered statistically significant.

## Results

### *Pcyt2* ^*+ /-*^ NASH genome is globally hypermethylated and promoters hypomethylated

**Fig 1:**
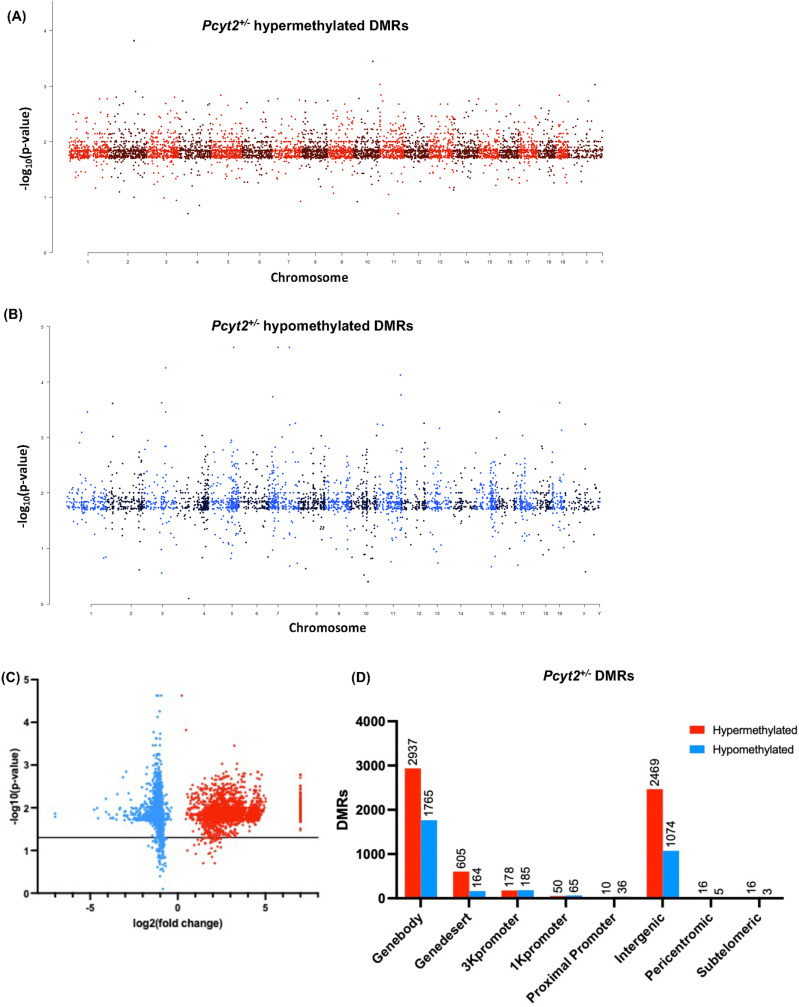
Epigenome-wide methylation profiles. Manhattan plots of differentially methylated regions (DMRs) across chromosomes shows that *Pcyt2*^* + /-*^ exhibits (A) 6281 hypermethylated and (B) 3297 hypomethylated DMRs. (C) Volcano plots show the magnitude of change in DMRs from in *Pcyt2*^* + /-*^ where blue and red dots represent hypo- and hypermethylation, respectively. DMRs above the black line are significant (*p* = 0.05). Data points at *x*=+/-7 represent genes where fold change = infinity. (D) The distribution of significant hyper- and hypomethylated DMRs in relation to the nearest gene region show that most DMRs are in the genebody, and the promoter region that is most effected is the 3K region.

To determine if altered DNA methylation accompanies the functional pathologies we observed previously in *Pcyt2*^* + /-*^ NASH liver [16], we conducted an epigenome-wide methylation analysis on whole livers from *Pcyt2*^ + / +^ and *Pcyt2*^* + /-*^ mice. Data are presented as *Pcyt2*^* + /-*^ relative to the wild-type, *Pcyt2*^ + / + ^. *Pcyt2*^* + /-*^ NASH liver genome contains 9578 differentially methylated CpG regions (DMRs), of which 6281 (65.6%) were hypermethylated and 3297 (34.4%) were hypomethylated (Fig. 1A, B and C). All DMRs were annotated based on genomic location and categorized by chromosome and genomic region (3K promoter, 1K promoter, 0.5K proximal promoter, gene body, gene desert, intergenic, pericentromic, subtelomeric regions) (Fig. 1D, Table S1). The gene body and intergenic regions contain the greatest number of total DMRs, and the 3K promoters contain the largest number of promoter-DMRs. All promoter regions are more hypomethylated than hypermethylated. In 3K, 1K and 0.5K promoters, the number of DMR hypomethylations is greater than the number of DMR hypermethylations by 2-fold, 2.5-fold, 6.8-fold, respectively, relative to total DMRs.

### Distribution of *Pcyt2* ^*+ /-*^ NASH differently methylated genes

**Fig 2:**
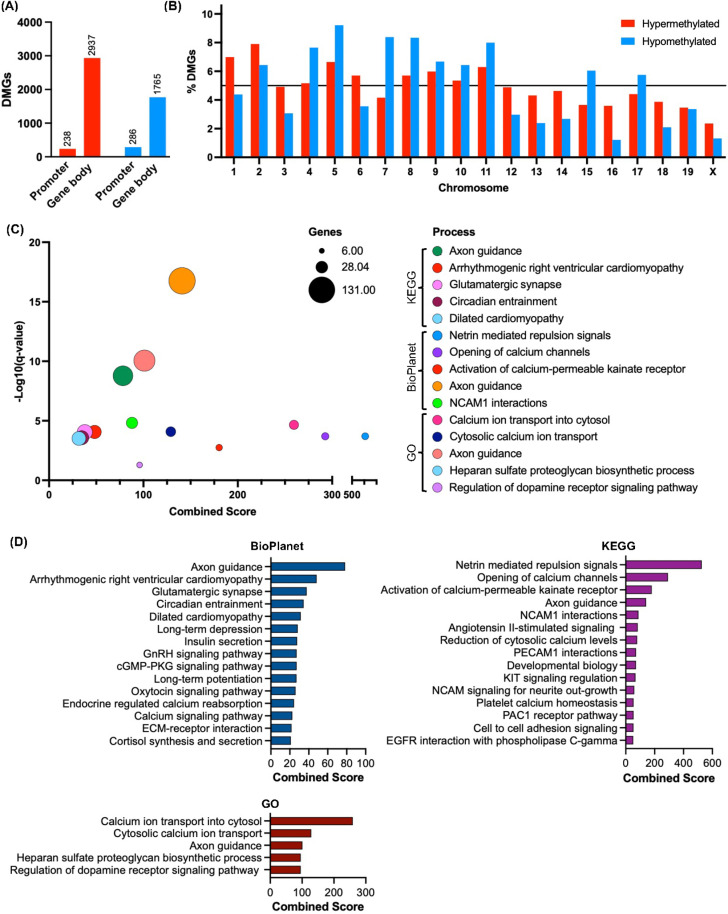
Genomic distribution and functional enrichment of differentially methylated genes. DMRs present in gene-coding regions, termed differentially methylated genes (DMGs), were selected for further analysis. (A) *Pcyt2*^* + /-*^ contains 5226 wherein for hypermethylated DMGs, 7.5% are in promoter regions and 92.5% are in the gene body, and in hypomethylated DMGs, 14% are in promoter regions and 86% in the gene body. (B) The chromosomal distribution of hyper- and hypomethylated DMGs shows that 1, 2 and 5 harbour the greatest number of DMGs. Bars above the black line show chromosomes possessing DMGs that account for over 6% of the total DMGs. Top 5 (C) and top 15 (D) functionally enriched pathways of hyper- and hypomethylated DMGs in *Pcyt2*^* + /-*^ using BioPlanet, Gene Ontology (GO) Biological process and Kyoto Encyclopedia of Genes and Genomes (KEGG) databases. Functional enrichment shows an enrichment in pathways linked well-known NASH development processes such as to hepatic inflammation and fibrosis, and interestingly, a great enrichment in nervous system related pathways suggesting the importance of phospholipid homeostasis in CNS/PNS function. Analysis was completed using a Benjamini-Hochberg adjusted *p* value (<0.05) as the significance cut off. Combined score was represented using a z-score permutation background correction on Fisher’s exact test p-value to generate an overall score for each pathway; this method of pathway analysis has been shown to recover the most correct terms [31].

DMRs located in the protein-coding and regulatory promoter regions (gene body and 0.5K, 1K and 3K upstream) were selected for further analysis and referred as differently methylated genes (DMGs). The *Pcyt2*^* + /-*^ NASH liver contains 5226 DMGs of which 3175 are hypermethylated (61%) and 2051 (39%) hypomethylated (Fig. 2A). In hypermethylated DMGs, 7.5% are in promoter regions and 92.5% are in the gene body, whereas in hypomethylated DMGs, 14% are in promoter regions and 86% in the gene body. Hypermethylated DMGs were more uniformly distributed across chromosomes than hypomethylated DMGs, with the proportions of chromosomal DMGs relative to total DMGs ranging from 2.36-7.9% (hypermethylated) and 1.2-9.2% (hypomethylated). The largest proportions of hypermethylated DMGs were located at Chromosomes 1, 2, 5, and 11 (6.3-7.9%), whereas there were greater concentrations of hypomethylated DMGs that were located at Chromosomes 5, 7, 8, and 11 (7.9-9.2%) (Fig. 2B).

### Differentially methylated genes belong to key pathways involved in NASH pathogenesis

To explore the biological significance of DMGs we performed pathway enrichment analysis using KEGG, BioPlanet, and Gene Ontology (GO): Biological Process databases. Because methylation within either the promoter and gene body regions can often produce opposite effects on gene expression, we grouped DMGs based on the location of methylated CpGs (promoter or gene body) and methylation status (hyper- or hypomethylated) and analyzed groups separately to determine how this may have influenced the total pathway analysis. The affected pathways were similar across hyper- and hypomethylated regions, therefore we analyzed them together. The top 5 significantly enriched terms from each database are shown in Fig. 2C. The most significantly enriched methylation pathways are nervous system related processes (Netrin mediated repulsion signals and Axon guidance) and calcium signalling processes (Opening of calcium channels triggered by depolarization of the presynaptic terminal, Activation of calcium-permeable kainate receptor, calcium ion transport into cytosol).

Top 15 enriched pathways in individual databases are shown in Fig. 2D and Table S2. Overall, they identify processes related to Growth and Development Signaling (Axon guidance, Developmental biology, KIT signaling regulation, NCAM signaling for neurite out-growth, Angiotensin II-stimulated signaling, Regulation of dopamine receptor signaling pathway) and Neurotransmission and synaptic plasticity (Glutamatergic synapse, Long-term depression, Long-term potentiation, cGMP-PKG signaling pathway) are the most significantly enriched processes. Processes relating to metabolism and hormonal regulation (Insulin secretion, GnRH signaling pathway, Cortisol synthesis and secretion, Endocrine regulated calcium reabsorption, Oxytocin signaling pathway, PAC1 receptor pathway) and ECM and cell adhesion (ECM-receptor interaction, NCAM1 interactions, PECAM1 interactions, Cell to cell adhesion signaling, EGFR interaction with phospholipase C-gamma, Heparan sulfate proteoglycan biosynthetic process) are also among the top 15 significantly enriched processes (Table S2), which are linked to NASH [35–37].

### DMEGs that are differently expressed in asymptomatic young *Pcyt2* ^*+ /-*^ mice

**Fig 3:**
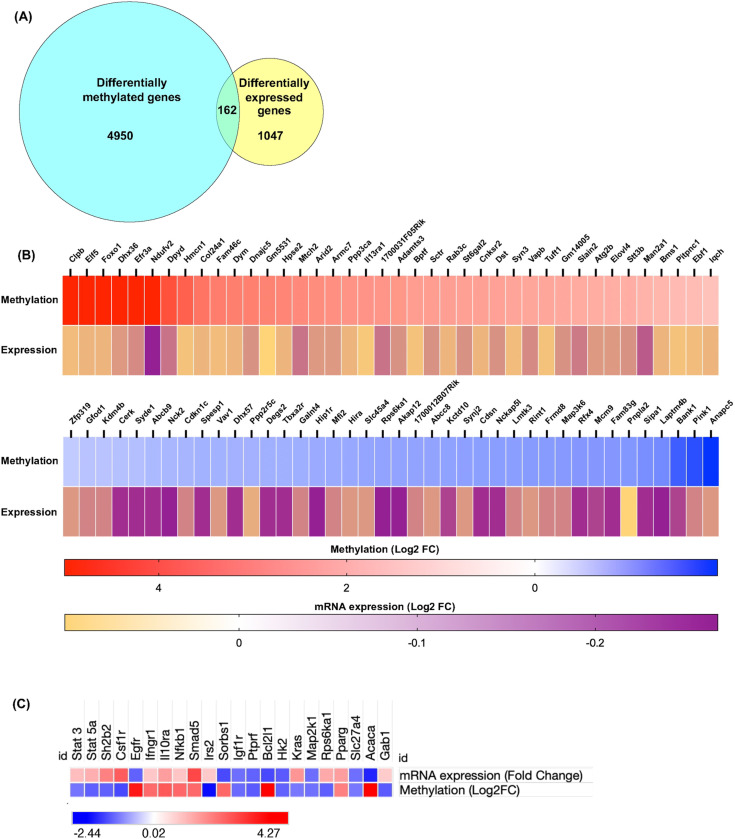
DNA methylation associates with altered mRNA expression of key genes involved in NASH pathogenesis. (A) Venn diagram showing the overlapping genes from *Pcyt2*^* + /-*^ Me-DNA-seq and 2-month *Pcyt2*^* + /-*^ liver microarray (GEO# GSE55617), showing the set of genes that are both differentially methylated and expressed. (B) Heatmaps show the top 20 hypermethylated and hypomethylated genes based on mRNA change in 2-month-old *Pcyt2*^* + /-*^ mice. Genes that exhibited that greatest changes in methylation and mRNA expression including genes related to mitochondrial function, autophagy, lipid metabolism, glucose homeostasis, MAPK signalling, inflammation, n-glycan biosynthesis, and cell cycle. (C) RT-PCR analysis of insulin, MAPK and JAK/STAT signalling pathways in 8-month-old *Pcyt2*^* + /-*^ mice shows altered DNA methylation influences the expression of genes within key pathways relevant to NASH pathogenesis. Genes that have a methylation increased or decreased of infinity are represented as Log2FC = 5.

Next, we wanted to determine the differentially methylated genes that have altered mRNA expression. To do this, we performed an integrated analysis of Me-DNA-seq data with microarray and RT-PCR array expression data to determine genes that are both differentially methylated and differentially expressed. We have previously established that young *Pcyt2*^* + /-*^ mice (2-mo) show early changes in gene expression that contributed to the adult development of NASH, including dysregulation of genes and pathways involved in fatty acid oxidation, triglyceride and ammonia metabolism, hepatic steatosis and abnormal liver physiology [15,16]. To determine the genes that are epigenetically modified as a direct effect of gene knockout, rather than a consequence of the adult diseased state, we overlapped of *Pcyt2*^* + /-*^ Me-DNA-seq data with 2-month *Pcyt2*^* + /-*^ liver microarray (GEO# GSE55617) [16] and found 162 differentially methylated and expressed genes (DMEGs) (Fig. 3A). Heatmaps show hypermethylated and hypomethylated DMEGs with the greatest changes in mRNA expression (Fig. 3B, Table S3). These included genes related to mitochondrial function (*Ndufv2*, *Mtch2, Pink1*), autophagy (*Atg2b)*, lipid metabolism (*Elov4*, *Pitpnc1* and Atgl lipase gene *Pnpla2*,), glucose homeostasis (*Foxo1*), MAPK signalling (*Map3k6*, *Rps6ka1*), inflammation (*Il13ra1*), n-glycan biosynthesis (*Man2a1, Sta6gal2),* collagen metabolism *(Adamts3, Dst, Col24a1)* and cell cycle (*Ppp2r5c, Cdkn1c Anapc5*, *Rint1, Vav1, Nck2*). Importantly, these findings complement our previous work showing that young *Pcyt2*^* + /-*^ mice exhibit early defects in mitochondria function and energy metabolism at the mRNA and protein levels and indicate that pathological processes which culminate in the adult onset of NASH also epigenetically initiated in young mice.

### DMEGs that are differently expressed in *Pcyt2* ^*+ /-*^ NASH

We have previously shown that *Pcyt2*^* + /-*^ insulin resistance and NASH develop at 6-8-mo [16]. To identify DMEGs associated with NASH, we overlapped *Pcyt2*^* + /-*^ Me-DNA-seq data with the mRNA expression data obtained for insulin [16], MAPK and JAK/STAT signalling pathways in 8-month *Pcyt2*^* + / −*^ liver. The heatmap (Fig. 3C, Table S4) identified DMEGs in several NASH modulated genes from the insulin/glucose metabolism (*Irs2, Ptprf, Hk2, Sorbs1*), fatty acid metabolism (*Slc27a4, Acaca*), MAPK signalling (*Kras, Map2k1, Rps6ka1*), JAK/STAT signalling (*Stat3, Stat5a, Sh2b2, Csf1r, Egfr),* inflammation (*Ifngr1, Il10ra, Nfkb1*), cell growth (*Igf1r, Pparg, Smad5, Gab*), and apoptosis (*Bcl2l1*).

### Protein interaction network reinforces *Pcyt2* ^*+ /-*^ phenotype

**Fig 4:**
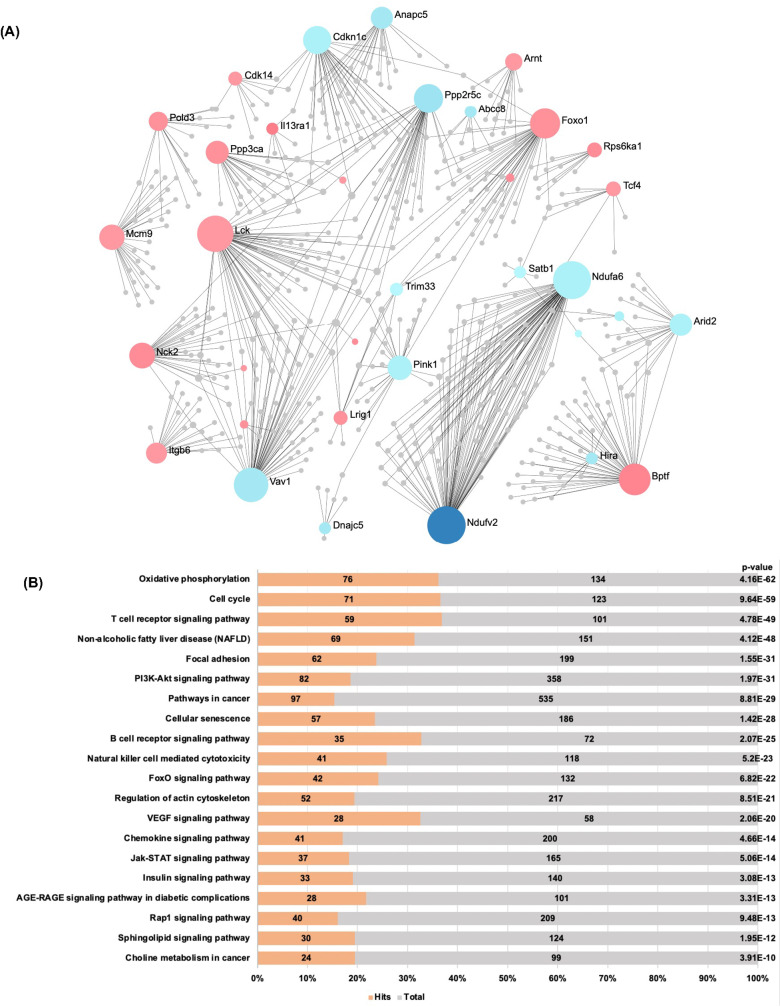
Network analysis of genes that are both differentially methylated and expressed (DMEG). (A) Protein-protein interaction network of DMEGs with one large subnetwork consisting of 34 interrelated seed genes, 540 nodes and 686 edges. Important genes that show the greatest degree of connectively and thus, the greatest influence on the network include proteins related to mitochondrial function, glucose homeostasis, and regulation of cell cycle and proliferation. Red and blue nodes indicate genes with increased and decreased mRNA expression, respectively, and circle size indicates the grade of connectivity. (B) Functional enrichment analysis of protein-protein interaction network using KEGG database showing and enrichment in processes highly relevant to including Pi3k-Akt signalling pathway, Foxo signalling pathway, insulin signalling, oxidative phosphorylation, multiple inflammation related pathways and NAFLD and previously unexplored pathways such as regulation of actin cytoskeleton, focal adhesion, cell cycle regulation and cellular senescence.

Next, we probed the connections between the above set of genes that were both differentially methylated and expressed. Using the STRING database, we constructed a protein-protein interaction network of the overlapped *Pcyt2*^* + /-*^DMEGs from Me-DNA-seq data with the genes from the microarray and RT-PCR array expression data. There were 184 total overlapping genes that formed one large subnetwork consisting of 34 interrelated seed genes, 540 nodes and 686 edges (Fig. 4A), and multiple smaller subnetworks (Fig S1A-D). The nodes with the highest degree centralities include genes encoding mitochondrial proteins Ndufv2, Ndufa6 and Pink1; glucose homeostasis related Foxo1, T-/B-cell related Lck; and proteins implicated in the regulation of cell cycle and proliferation: Vav1, Ppp2r5c, Cdk14, Cdkn1c, Anapc5. The nodes with the highest betweenness centrality include Foxo1, Pink1 and Cdkn1c indicating these genes are key links within this network and thus, their regulation exerts the greatest influence on the network. Top 50 nodes based on degree centrality are in Table S5. Smaller subnetworks reveal connectivity between genes involved spliceosome and ribosome biogenesis, mucin type O-glycan biosynthesis and amino acid metabolism (Fig S1A-D).

KEGG analysis of protein network interactions from above reveals an enrichment in pathways that are highly relevant to the *Pcyt2*^* + /-*^ phenotype including Pi3k-Akt signalling pathway, Foxo signalling pathway, insulin signalling, oxidative phosphorylation, multiple inflammation related pathways, and NAFLD. All of these pathways are supported by our previous evidence showing molecular perturbations to these pathways, and the physiological consequences including reduced fatty acid oxidation, deficient insulin signalling, liver inflammation and steatosis in *Pcyt2*^* + /-*^ mice [15,16] (Fig. 4B). In addition, this analysis highlighted previously unidentified pathways: cellular senescence and cell cycle regulation. These pathways include genes involved in autophagy, ubiquitin-mediated proteolysis, apoptosis the regulation of actin cytoskeleton, focal adhesion, and many directly involved in the cell cycle including MAPK signalling, cyclin-dependent kinases, cyclins, anaphase promoting complex subunits and G1 to S cell cycle regulators (Table S6). Together this analysis highlights the impact of epigenetic regulation on the transcriptome and shows that these differentially methylated and expressed genes belong to key pathways involved the development of NASH, emphasising the importance of their dysregulation in the pathological development of the *Pcyt2*^* + /-*^ phenotype.

### Transcriptional overlap with alternate NASH expression profile

Finally, to relate our findings to other models of NASH and further investigate candidate genes that could serve predictive value, we compared *Pcyt2*^* + /-*^ DMEGs to a publicly available microarray dataset (GDS4883) of murine NASH caused by a methionine- and choline-deficient/high fat diet (MCD+HF). We identified 15 genes (Table S7) including the cell cycle (*Cdk14*, *Cdk17* and *Anapc5*) and mitochondrial (*Tmem65*, *Mtch2,* and *Pccb)* genes as common genes contributing to NASH development in these models. The same cell cycle genes (*Cdk14*, *Cdk17* and *Anapc5*) show aberrant methylation and expression in both young and adult *Pcyt2*^* + /-*^, and the alternative NASH model (methionine/choline-deficient/high fat diet), indicating their strong relevance to NASH development and as potential prognostic indicators.

### PEA treatment dramatically reduces total aberrant DNA methylation

**Fig 5:**
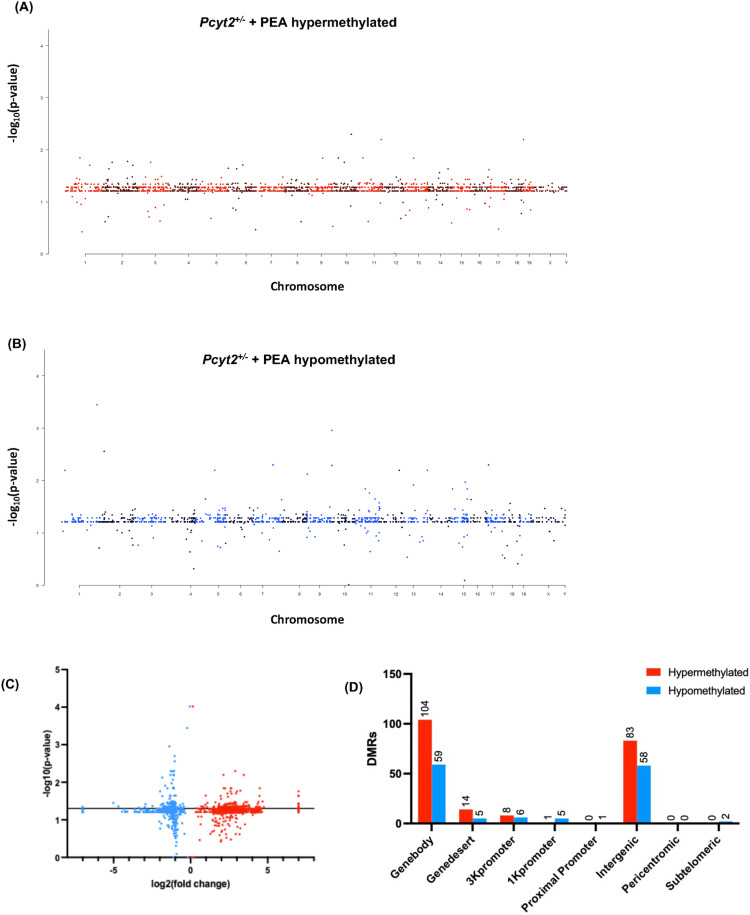
Epigenome-wide methylation profiles. Manhattan plots of differentially methylated regions (DMRs) across chromosomes shows that PEA treatment equally reduces both hyper-and hypomethylated DMRs from (A) 6281 hypermethylated DMRs in *Pcyt2*^* + /-*^ to 210 in *Pcyt2*^* + /-*^ + PEA and (B) 3297 hypomethylated in *Pcyt2*^* + /-*^ to 136 hypomethylated in *Pcyt2*^* + /-*^ + PEA. (C) Volcano plots show the magnitude of change in *Pcyt2*^* + /-*^ + PEA DMRs relative to *Pcyt2*^* + / + *^. Blue and red dots represent hypo- and hypermethylation, respectively. DMRs above the black line are significant (*p* = 0.05). Data points at *x*=+/-7 represent genes where fold change = infinity. Both the Manhattan and volcano plots show dramatically reduced DNA methylation in PEA treated liver. (D) The distribution of significant hyper- and hypomethylated DMRs in relation to the nearest gene region in *Pcyt2*^* + /-*^ + PEtn show that most DMRs are in the genebody, and the promoter region that is most effected is the 3K region.

We have previously shown that an 8-week treatment with the artificial Pcyt2 substrate PEA prevents and/or reverses multiple aspects of the *Pcyt2*^* + /-*^ NASH phenotype including liver steatosis, inflammation, and the protein levels of several glucose and fatty acids metabolic regulators [16]. Here, we find aberrant DNA methylation of *Pcyt2*^* + /-*^ NASH liver with an enrichment in pathways related to NASH pathogenesis (Figs. 1-4). Therefore, we performed an epigenome-wide DNA methylation analysis on the livers from *Pcyt2*^* + /-*^ mice treated with PEA (*Pcyt2*^* + /-*^ + PEA) to determine whether the amelioration of phenotypic symptoms was accompanied by a shift in DNA methylation to a profile that more closely resembles that of *Pcyt2*^* + / + *^. Indeed, PEA treatment reduced total DMRs from 9578 in *Pcyt2*^* + /-*^ to 346 in *Pcyt2*^* + /-*^* + *PEA (96.4%) with hypermethylated DMRs reduced from 6281 to 210 (96.7%) and hypomethylated DMRs reduced from 3297 to 136 (95.9%) (Fig. 5A-C, Table S8).

To determine how PEA altered the genomic distribution of hypermethylated and hypomethylated DMRs, a χ^2^ test for independence was used to compare their relative proportions across genomic regions of *Pcyt2*^* + /-*^ and *Pcyt2*^* + /-*^ + PEA using *Pcyt2*^* + /-*^ as the null model (Fig. 5D, Table S9). The overall χ^2^ value revealed a change in the distribution of DMRs after PEA treatment that is predominantly driven by shifts in the hypomethylations of the gene body, intergenic region and 1K promoter (*p* < 0.0001). Relative to other regions, PEA induced a greater reduction of gene body hypomethylation. The percentage of hypomethylated DMRs in the gene body decreased from 54% in *Pcyt2*^* + /-*^ to 43% in *Pcyt2*^* + /-*^ + PEA while hypomethylated DMRs in intergenic and 1K promoter regions increased from 33% to 43% and from 2% to 3.7%. On the other hand, PEA treatment reduced hypermethylated DMRs more evenly across genomic regions. Together this shows that PEA treatment dramatically attenuated *Pcyt2*^* + /-*^ DMRs but unequally affected hypermethylation and hypomethylation patterns. There was a greater attenuation of total hypermethylation and gene body hypomethylation with a shift in the genomic distribution of hypomethylated DMRs.

### PEA treatment strongly attenuates the NASH altered methylation of protein coding regions

**Fig 6:**
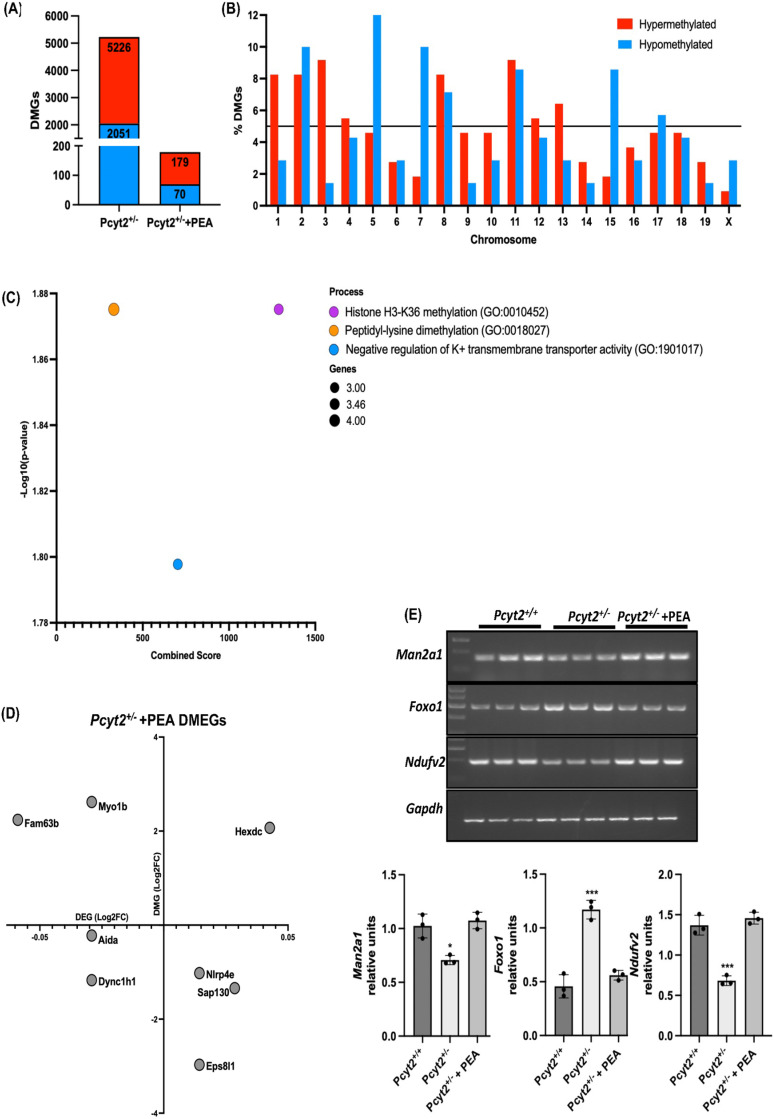
Genomic distribution and functional enrichment of differentially methylated genes. (A) PEA treatment reduced *Pcyt2*^* + /-*^ DMGs by 96.7% from 5226 to 175 DMGs in *Pcyt2*^* + /-*^ + PEA with 43% and 57% hyper- and hypomethylated DMGs, respectively, this shows a similar distribution to *Pcyt2*^* + /-*^ showing a coordinated action of methylation and demethylation processes. (B) DMG distribution across chromosomes showing the highest relative proportions of hypermethylated genes PEA at Chromosomes 1, 2, 3, 8, 11 and 13 (6.4-9.2%) and the highest proportions of hypomethylated genes at Chromosomes 2, 5, 7, 8, 11, and 15 (7.1-14%). (C) Enrichment analysis of DMG in *Pcyt2*^* + /-*^ + PEA mice showing nearly all enriched pathways were abolished by PEA treatment (D) PEA treatment reduced genes that are differently methylated and expressed (DMEGs) by 95.2% from 162 to 8. Correlation plot of the remaining 8 DMEGs after PEA treatment. Only one gene, *Aida,* was differentially methylated in promoter region. **(E)** PCR validation of key genes that were differentially methylated and differentially expressed (*Man2a1*, *Foxo1* and *Ndufv2*) showing that the expressions of all genes were attenuated by PEA treatment. Band intensities were measured using ImageJ. * p <  0.05, ***p <  0.001.

DMRs located in the protein coding gene body or promoter regions (DMGs) were the focus for further analysis. PEA treatment of *Pcyt2*^* + /-*^ reduced *Pcyt2*^* + /-*^ DMGs by 96.7%, from 5226 in *Pcyt2*^* + /-*^ to 175 in *Pcyt2*^* + /-*^ + PEA (Fig. 6A). In *Pcyt2*^* + /-*^ the ratio of hyper- and hypomethylated DMGs is different than in total DMRs with 45% and 55% hyper- and hypomethylated DMGs, respectively. This ratio was maintained after PEA treatment with 43% and 57% hyper- and hypomethylated regions, showing a coordinated action of methylation and demethylation processes. When comparing the distribution of DMGs across *Pcyt2*^* + /-*^ and *Pcyt2*^* + /-*^ + PEA chromosomes (Fig. 2B and 6B, Table S10), *Pcyt2*^* + /-*^ + PEA shows a similar distribution. The highest relative proportions of hypermethylated genes in *Pcyt2*^* + /-*^* + *PEA are at Chromosomes 1, 2, 3, 8, 11 and 13 (6.4-9.2%), and for hypomethylated genes, at Chromosomes 2, 5, 7, 8, 11, and 15 (7.1-14%).

Nearly all enriched pathways in *Pcyt2*^* + /-*^ NASH were abolished by PEA treatment. Pathway analysis of the 175 DMGs *Pcyt2*^* + /-*^ + PEA showed only 3 enriched pathways: histone H3-K36 methylation, peptidyl-lysine demethylation and negative regulation of potassium transporter (Fig. 6C, Table S11). PEA specifically affected the aberrant methylation of key epigenetic regulators, including DNA methylation genes, histone methylation/demethylation, histone deacetylation, methyl binding proteins (which recruit other epigenetic modifiers), and largely attenuated the hypermethylation of *Setd2* and hypomethylation of *Kmt2d*. Together these data show that PEA treatment prevents or reverses the aberrant hyper- and hypomethylation of genes in *Pcyt2*^* + /-*^ that likely contributed to the development of NASH. These findings complement our previous work showing that PEA treatment reversed multiple phenotypic aspects of *Pcyt2*^* + /-*^ NASH [16].

### PEA nearly abolishes the expression of differently methylated genes

PEA treatment produced a dramatic transcriptional effect and reduced the number of DMEGs by 95.2% from 162 to 8. The remaining DMEGs include: *Hexdc*, *Sap130, Nlrp4e* and *Eps8l1* (increased mRNA expression), and *Fam63b*, *Myo1b, Aida* and *Dync1h1* (decreased mRNA expression) (Fig. 6D). Only one gene was differentially methylated in the promoter region, *Aida,* and showed decreased methylation and mRNA expression. Identification of these genes provides mechanistical clues to better understand our previous finding that PEA treatment attenuates but does not completely prevent *Pcyt2*^* + /-*^ NASH. For example, *Aida* deficiency causes severe obesity, hypertriglyceridemia and increased intestinal lipid absorption in mice [38]; thus, the failure of PEA to reverse the aberrant methylation and expression of *Aida* may impede the full resolution of the *Pcyt2*^* + /-*^ phenotype. To validate these findings, we performed PCR analysis on key genes that were differentially methylated and differentially expressed (*Man2a1*, *Foxo1* and *Ndufv2)* and we show that all were attenuated by PEA treatment (Fig. 6E). These data indicate that the aberrant epigenetic pathways contributing to *Pcyt2*^* + /-*^ NASH were reversed by PEA treatment, likely in part through the restoration of methylation status of key DNA/histone methylation enzymes and hypermethylation of histone methyltransferases *Prdm9, Ash1l*. Together this shows the transcriptional significance of the reversal in *Pcyt2*^* + /-*^ methylation patterns and indicates potential targets for future studies.

### PEA distinctly epigenetically regulates groups of genes

Our previous study showed that while PEA treatment attenuated multiple aspects of *Pcyt2*^* + /-*^ NASH, it was unable to completely reverse all aberrant mRNA/protein expression [16]. Therefore, we were interested in further examining the DMGs that were not reversed by PEA treatment to better understand the role that these genes may play in the persistence of the *Pcyt2*^* + /-*^ pathologies. There are 114 DMGs that are not reversed by PEA treatment whereby the methylation status is either incompletely reversed, unresponsive, or exacerbated by PEA. These DMGs are involved in processes such as axon guidance, netrin signalling, notch signalling, n-glycan biosynthesis, regulation of actin cytoskeleton, focal adhesion, MAPK signalling and sphingolipid metabolism (Fig S2). Several of these processes such as axon guidance, netrin signalling and adhesion signalling, are common with the enriched pathways in *Pcyt2*^* + /-*^ without PEA treatment, but do not include the same genes, suggesting an influence of PEA on these pathways that may specifically contribute to NASH reversion.

**Fig 7:**
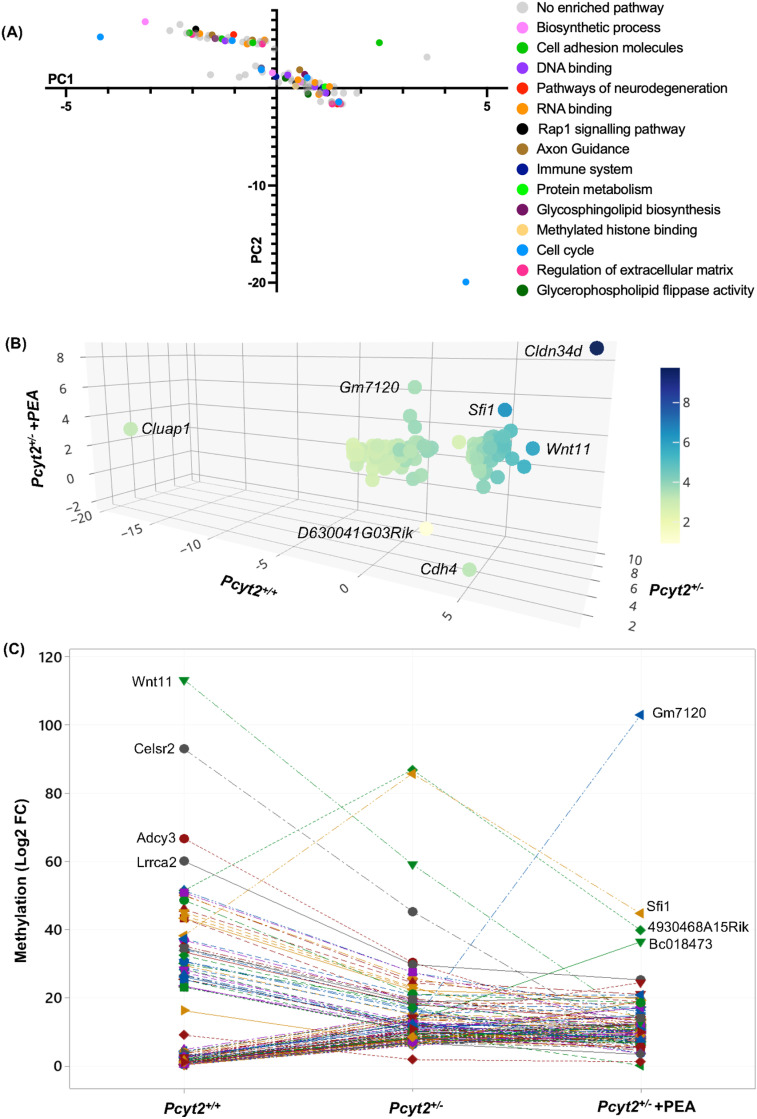
The effect of PEA supplementation on DMGs that were not reversed. (A) Principal component analysis of the methylation changes of the DMG’s that were not revered by PEA treatment showing 2 central clusters of genes. Genes were assigned grouping variables according to known associations with cellular pathways. Genes involved in processes such as glycerophospholipid flippase activity, DNA binding and regulation of extra cellular matrix activity were regulated similarly. In contrast, genes involved in RNA binding and cell cycle showed a greater difference in epigenetic regulation. (B) 3D plot showing the 114 common DMGs and specific DMGs that exhibit distinct separation and therefor different regulation from 2 central clusters. Data presented as log2 fold change. Colour indicates variation along the y-axis (*Pcyt2*^* + /-*^). (C) Line plot visualizing the directional change across *Pcyt2*^* + / + *^, *Pcyt2*^* + /-*^ and *Pcyt2*^* + /-*^ + PEA, with Key genes that exhibit the greatest exacerbation or attenuation of methylation status by PEA treatment are labelled. Most DMGs in this group were unaffected by PEA showing that methylation changes at these loci in *Pcyt2*^* + /-*^ are strong and not reversible. The subset of genes that showed an exaggeration of methylation status should be targets of future studies to delineate the therapeutic potential of PEA.

To better understand the regulation of the DMGs that were not reversed with PEA treatment, we performed a principal component analysis (PCA) of the methylation changes of these DMGs across *Pcyt2*^* + / + *^, *Pcyt2*^* + /-*^ and *Pcyt2*^* + /-*^* + *PEA. The PCA shows that most of the variation was represented in the first and second component, accounting for 60.2% and 28.5% of the variation, and formed two central gene clusters (Fig. 7A, Fig S3A-F). To determine the regulated pathways, the PC1 and PC2 cluster genes were analysed based on KEGG or GO:Molecular Function pathways. Glycerophospholipid flippase activity, DNA binding, and Regulation of extra cellular matrix genes clustered together while the genes belonging to RNA binding and Cell cycle showed some separation in PC1 and PC2, indicating distinct regulation (Table S12). Several specific DMGs exhibited apparent separation from the groups (*Cluap1, Sfi1, Wnt11, Cdh4, Gm7120, Cldn34d, D630041G03Rik)*, showing that these specific genes exhibit unique methylation patterns across *Pcyt2*^* + / + *^, *Pcyt2*^* + /-*^ and *Pcyt2*^* + /-*^* + *PEA (Fig. 7B).

### PEA strengthens the methylation status in a subset of differently methylated genes

Finally, the line plot visualizes the changes in methylation across *Pcyt2*^* + / + *^, *Pcyt2*^* + /-*^ and *Pcyt2*^* + /-*^ + PEA (Fig. 7C). This shows that the majority of the DMGs that continue to persist after PEA treatment were unaffected by PEA, showing that methylation changes at these loci in *Pcyt2*^* + /-*^ are strong and not reversible. For example, genes involved in DNA binding were strongly methylated in *Pcyt2*^* + /-*^ and their methylation levels remained the same in *Pcyt2*^* + /-*^* + *PEA, showing that these changes were not reversed. On the other hand, the line plot highlights key DMGs that were most affected by PEA treatment including *Wnt11, Celsr2, Adcy3, Lrrca2, Sfi1, Gm7120* (predicted to be an integral membrane component)*, Cldn34d* (predicted to be involved in cell adhesion and membrane function), and *Bc018473* (unknown function) (Fig. 7C, Fig S2). The greatest increases in methylation in *Wnt11*, *Celsr2* and *Adcy3* could indicate future routes of investigation to elucidate the potential of PEA in NASH treatment, especially given their associations with obesity and diabetes [38–41].

Lastly, there is a subset of DMGs where the altered methylation in *Pcyt2*^* + /-*^ was exacerbated by PEA treatment. For example, several cell adhesion genes are hypomethylated in *Pcyt2*^* + /-*^ and further hypomethylated in *Pcyt2*^* + /-*^* + *PEA, showing that PEA strengthens the epigenetic status in these genes. Likewise, several CNS development genes are hypermethylated in *Pcyt2*^* + /-*^ and further hypermethylated by PEA treatment. Together this shows that there are subsets of epigenetically dysregulated genes in *Pcyt2*^* + /-*^ that are either unresponsive to PEA treatment or where PEA reinforces the hypo- or hypermethylation present at these genes. These findings may suggest important targets for future studies to better understand mechanistic underpinnings of NASH and to explore the potential of PEA as a therapeutic intervention.

## Discussion

Early disruptions in hepatic gene and protein expression exist prior to overt disease onset in both *Pcyt2*^* + /-*^ mice [15,16,24], and humans [42], yet the molecular mechanism underpinning the initiation, progression and heterogenous outcomes of NASH are unresolved. Delineating DNA methylation changes during NASH development can facilitate improved diagnostic and therapeutic strategies. In our previous study, we showed that Pcyt2 deficiency induces NASH characterized by hyperglycemia, increased glycogen and TAG accumulation, inflammation, fibrosis, and obesity [16]. Importantly, the *Pcyt2*^* + /-*^ mouse model recapitulates both the metabolic and histopathological features of NASH, in which pathologies develop with age, like humans. In addition, we showed that treatment with the artificial Pcyt2 substrate PEA attenuated several aspects of the *Pcyt2*^* + /-*^ NASH, including lipid accumulation, fibrosis, and the inflammatory response. Here, we identify significant DNA methylation changes in *Pcyt2*^* + /-*^ NASH and show that an 8-week PEA treatment dramatically reverts these aberrant methylation patterns.

The *Pcyt2*^* + /-*^ liver is substantially differentially methylated relative to *Pcyt2*^* + / + *^, showing hyper- and hypomethylation across all chromosomes and protein coding regions, consistent with our finding that the *Pcyt2*^* + /-*^ liver exhibits altered gene and protein expression underlying its diseased state [15,16]. Specifically, the *Pcyt2*^* + /-*^ liver shows altered methylation of key genes involved in DNA and histone epigenetic modification including DNA methylation and demethylation, histone deacetylation and DNA methyl binding proteins which recruit and coordinate with other epigenetic modifiers. Outside of protein coding regions, *Pcyt2*^* + /-*^ liver exhibited vast differential methylation of non-coding intergenic regions. Among other DNA sequences, intergenic regions contain viral and transposable elements that are mostly silenced by bulk DNA methylation. Ectopic expression of these elements can lead to gene disruption, DNA mutations, compromise genomic stability and influence age-associated disease progression [43]. Specifically in the mouse genome, the loss of DNA methylation in intergenic regions due to *Dmnt1* insufficiency disrupts genomic integrity and leads to transcriptional reactivation of aggressive transposons in that causes deleterious biological consequences [44]. The *Pcyt2*^* + /-*^ NASH shows hypomethylation of intergenic regions, and hypomethylation of *Dmnt1* at 2 locations within its gene body. Associations between aberrant methylation of intergenic regions and T2DM [45], liver fibrosis [46] and hepatocellular carcinoma [47] have recently been established, suggesting the regulation of intergenic elements during *Pcyt2*^* + /-*^ NASH as an interesting area in subsequent studies.

We have previously shown that *Pcyt2*^* + /-*^ mice exhibit changes in liver metabolic regulators from young age but do not clinically manifest metabolic disease and NASH until adulthood [15,16]. Specifically, mitochondrial regulators and fatty acid metabolism proteins are defective in young mice and persist into adulthood, and here, we show that genes involved in these pathways including mitochondrial function (*Ndufv2*, *Pink1*, *Mtch2*), lipid metabolism (*Pnpla2*, *Pitpnc1*), glucose homeostasis (*Foxo1*) exhibit the most drastic changes in methylation and expression, and exert the greatest connectively within the protein interaction network indicating these as important epigenetically regulated genes in *Pcyt2*^* + /-*^ NASH. Functional enrichment of DMEGs and protein-protein interaction network analyses aligned with our previous protein and gene expression and physiological findings [15,16], showing an enrichment in hepatic fibrosis, PI3K-Akt and Foxo signalling, oxidative phosphorylation, insulin signalling/secretion, NAFLD and T2DM pathways. These findings suggest that epigenetic regulation likely initiates early and enduring defects in these pathways and underlies many of our previously established mechanisms in NASH development. In addition, multiple nervous system pathways were enriched such as axon guidance, glutamatergic synapse, activation of calcium-permeable kainate receptor and opening of calcium channels triggered by depolarization of the presynaptic terminal. Accumulating evidence shows the essential role of the hepatic autonomic nervous system and neurotrophic factors in energy homeostasis, liver injury and repair, and NASH pathogenesis [48–50], suggesting the potential importance of dysregulation of these pathways in the *Pcyt2*^* + /-*^ liver. The calcium signaling pathway regulates liver functions like bile secretion and glucose metabolism, and dysregulation of the insulin secretion pathway is associated with NAFLD and type 2 diabetes [51].

A new area highlighted by this study is cell cycle regulation and cellular senescence, indicating it relevance to age-depended NASH development. There is a significant enrichment in both the differential methylation and expression of cell cycle related genes including those that directly control cell cycle progression, focal adhesion, actin cytoskeleton, calcium signalling, ubiquitin-mediated proteolysis, autophagy, and apoptosis. Senescent cells are a hallmark of aging [52] and age-related diseases including obesity, T2DM, and fibrotic diseases [53,54], and triggered by various stressors shown in *Pcyt2*^* + /-*^ NASH (mitochondrial dysfunction, oxidative stress, inflammation, perturbed proteostasis and autophagy, epigenetic changes, and genomic instability) [55–57]. Moreover, in the *Pcyt2*^* + /-*^ liver PE synthesis and turnover is reduced [15,24], which can block cell cycle progression and induce senescence.

PE is directly involved with several important age-related processes, and the abundance of PE has been positively associated with longevity [58–63]. In *Pcyt2*^* + /-*^ mice PE synthesis and turnover are diminished, limiting the pool of PE available to participate in essential processes that are vulnerable to insufficient PE and impaired during aging [64–67]. The *Pcyt2*^* + /-*^ NASH genome-wide analysis in this study shows evidence for epigenetic alterations, deregulated nutrient-sensing, altered intercellular communication, mitochondrial dysfunction, and cellular senescence, all of which are classically defined hallmarks of aging [68]. Both hypo- and hypermethylation are associated with aging and the induction of genomic instability has been shown to accelerate aging in both mice and humans [69]. *Pcyt2*^* + /-*^ liver exhibits decreased expression of histone modifiers and other genes that regulate genomic integrity such as *Dhx36, Rint1* and H1 linker histone, *H1f0*. There is an early and sustained protein reduction in histone deacetylase Sirt1, which is increasingly being shown as an important gatekeeper of genomic stability and many age-related signalling pathways, and is diminished with aging [70,71]. Recently, accelerated aging was identified in muscle specific *Pcyt2*^* + /-*^ knockout model [72], suggesting the future investigation of regulation of *Pcyt2*^* + /-*^ liver aging during NASH development.

Aberrant methylation patterns in *Pcyt2*^* + /-*^ mice may result from the intersection of PE synthesis via CDP-ethanolamine pathway and one-carbon metabolism (which provides the methyl donor SAM). Limited PE synthesis in the *Pcyt2*^* + /-*^ liver, supresses PE and PC turnover to preserve total PE/PC levels [15,24,73], influencing one-carbon metabolism through SAM consuming PEMT activity and choline availability. This study showed decreased expression of genes involved in the one-carbon cycle (*Mat1a*, *Gnmt*, *Ahcy* and *Sds*,) and transsulfuration (*Cth* and *Cdo1)*, while previous analysis revealed decreased levels of plasma dimethylglycine [74], the product of betaine methylation of homocysteine by BHMT, an accumulation of glycine, and a reduction in serine, demonstrating systemic perturbations in one-carbon metabolism in *Pcyt2*^* + /-*^ mice. Together, this evidence suggests an overall perturbation to one-carbon cycling in *Pcyt2*^* + /-*^ liver that impacts methylation reactions to cause aberrant epigenetic modifications.

PEA treatment produced a dramatic effect on *Pcyt2*^* + /-*^ liver methylation patterns, reducing total *Pcyt2*^* + /-*^ DMGs by 96%. PEA is readily metabolized by the CDP-ethanolamine pathway [16] suggesting the possibility that PEA restored energy metabolism and normalized one-carbon cycling that prevented and/or reversed aberrant DNA methylation in *Pcyt2*^* + /-*^ NASH. PEA specifically restored the methylation status of key genes involved in epigenetic modifications including DNA methylation, histone methylation/demethylation, histone deacetylation, and methyl binding proteins that recruit other epigenetic modifiers.

Because PEA was not able to completely reverse *Pcyt2*^* + /-*^ NASH [16] and influences processes beyond the CDP-ethanolamine pathway [75,76], the subset of genes that exhibited elevated hyper-/hypomethylation of DMGs in response to PEA were examined as they may suggest pathways that affect NASH amelioration. For example, PEA induced the hypomethylation of *Adyc3* promoter and abolished the G protein alpha pathway which influences Adcy3 activity [77], a known therapeutic target for obesity [78]. Additionally, PEA induced the hypermethylation of *Celsr2* promoter and *Fam63b* in genebody, genes associated with blood lipids and T2DM [39,79], triglyceride, HDL and LDL phenotypes [80].

Importantly, nearly all DMGs that were also differentially expressed in *Pcyt2*^* + /-*^ were reversed to wild-type *Pcyt2*^* + / +*^ levels with PEA treatment, supporting our previous findings that PEA alters gene expression to ameliorate NASH. Like above, the 8 genes (*Hexdc*, *Sap130, Nlrp4e*, *Eps8l1*, *Fam63b*, *Myo1b, Aida* and *Dync1h*) that were not reversed by PEA treatment may be relevant targets for future studies. As previously mentioned, *Fam63b* and *Aida* play roles in triglyceride metabolism and are associated with metabolic phenotypes [38,80], suggesting their role in the persistence of *Pcyt2*^* + /-*^ NASH. Further, *Fam63b* and *Dync1h* may represent a link in pathologies outside of the liver. Altered DNA methylation patterns of *FAM63B* have been newly associated with bipolar disorder and schizophrenia [81,82]. Mutations in *DYNC1H1* gene have been associated with neurological diseases in humans [82] and here we show *Pcyt2*^* + /-*^ mice exhibit decreased methylation and expression. Interestingly, human patients harbouring loss of function mutations *PCYT2* have recently been found to develop complex hereditary spastic paraplegia (HSP) and a broad ataxia-spasticity condition and implicate disturbed neuronal phospholipid metabolism as a key pathological feature [83–85]. *Pcyt2*^* + /-*^ DMGs and DEGs exhibit disruptions to several neuronal related pathways, suggesting altered epigenetic regulation of nervous system function. Specifically, *Pcyt2*^* + /-*^ DMGs show altered regulation of dopamine receptor signalling pathway that was reversed by PEA which may be especially relevant given that parkinsonian-like symptoms are a frequent finding in HSP [86]. Importantly, PEA treatment was able to reverse nearly all neuronal and axonal DMGs.

## Conclusion

In this study we report that the *Pcyt2*^* + /-*^ liver methylome and transcriptome is altered and likely underlies much of the pathological development of NASH in *Pcyt2*^* + /-*^ mice. We show the therapeutic potential of PEA as it nearly completely reverses aberrant *Pcyt2*^* + /-*^ DNA methylation patterns by correcting the regulation of epigenetic modifiers. This analysis provides critical insight into NASH pathophysiology and the increasingly recognized association between epigenetic regulation and the development of age-related diseases. Characterizing DNA methylation changes associated with NASH development allows an improved mechanistic understanding of NASH progression and identifies potential diagnostic and prognostic biomarkers and therapeutic targets.

## Supporting information

S1 Fig (A-D) Smaller subnetworks of protein-protein interaction in DMEGs. *Pcyt2*^* + /-*^ NASH liver genome contains 9578 differentially methylated CpG regions (DMRs), with 65.6% hypermethylated and 34.4% hypomethylated DMRs were annotated based on genomic location and categorized by chromosome and genomic region (3K promoter, 1K promoter, 0.5K proximal promoter, gene body, gene desert, intergenic, pericentromic, subtelomeric regions).
(TIFF)

S2 Fig (A) Histogram of Pcyt2^ + /-^ + PEA DMGs that were not reversed by PEA treatment. The 15 enriched pathways include processes related to Growth and Development Signaling, Neurotransmission and synaptic plasticity, metabolism and hormonal regulation and ECM and cell adhesion, among others.
(TIFF)

S3 Fig A-F. PCA analysis of *Pcyt2*^ + /- ^ + PEA DMGs that were not reversed by PEA treatment. PCA of the methylation changes of these DMGs across *Pcyt2*^* + / + *^, *Pcyt2*^* + /-*^ and *Pcyt2*^* + /-*^* + *PEA shows that most of the variation was represented in the first and second component, accounting for 60.2% and 28.5% of the variation, and formed two central gene clusters.
(TIFF)

S1 File Supplementary Tables 1-12. Tables provide supplementary analyses of DMGs and DMEGs associated with Pcyt2 and Pcyt2 + PEA, including pathway enrichment, protein interactions, RT-PCR validation, chromosomal distribution, and statistical testing.
(ZIP)

S1 raw images Original gel images. (PDF)
