# Supplementary figures and images for: Epigenome-wide methylation analysis shows phosphonoethylamine alleviates aberrant DNA methylation in NASH caused by Pcyt2 deficiency
Source: PLoS One. 2025 Mar 28;20(3):e0320510. doi: 10.1371/journal.pone.0320510 (PMC11952270; doi:10.1371/journal.pone.0320510)

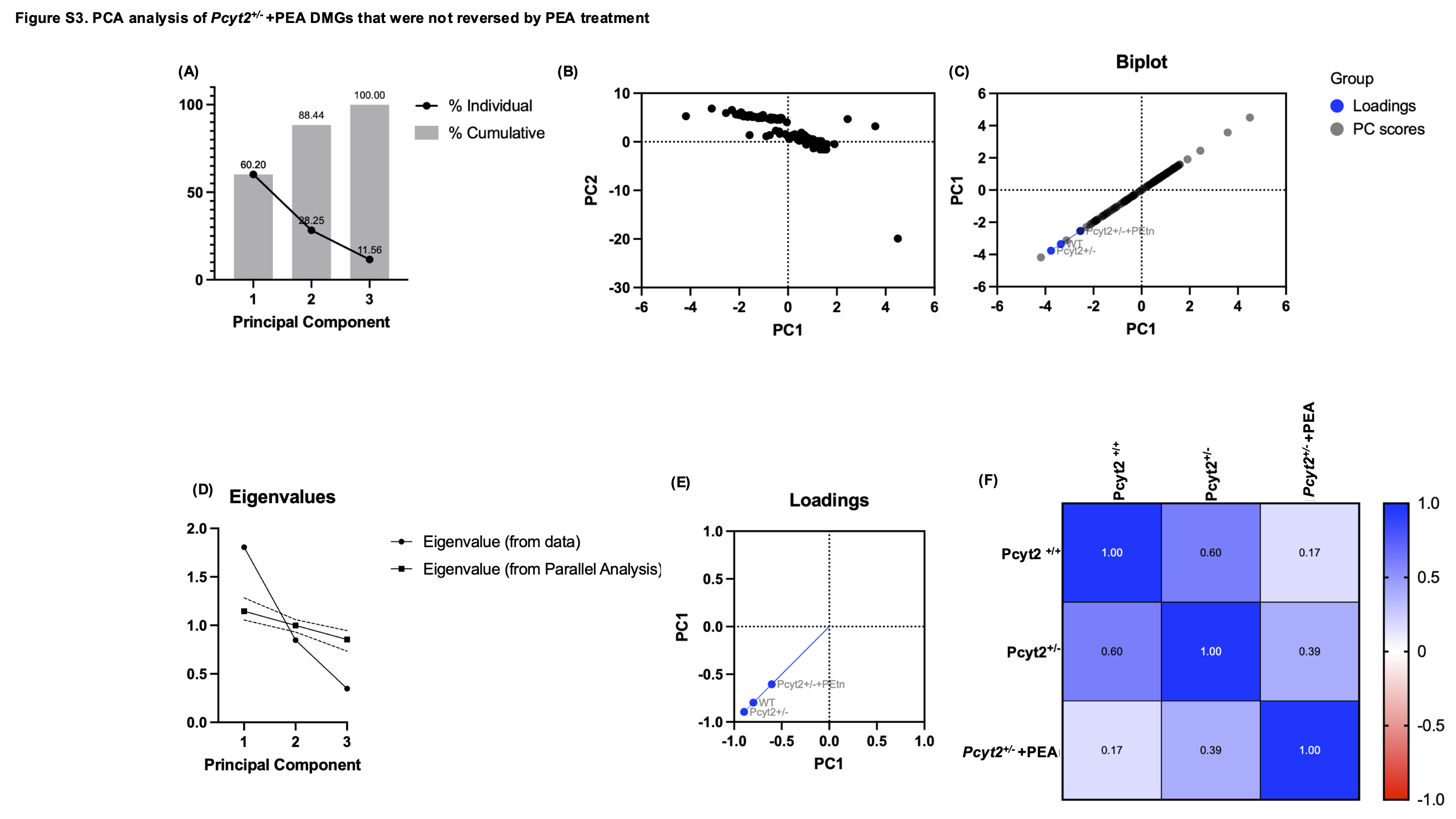

Supplement: S3 Fig — A-F. PCA analysis of Pcyt2 + /- + PEA DMGs that were not reversed by PEA treatment. PCA of the methylation changes of these DMGs across Pcyt2 + / + , Pcyt2 + /- and Pcyt2 + /- + PEA shows that most of the variation was represented in the first and second component, accounting for 60.2% and 28.5% of the variation, and formed two central gene clusters. (TIFF) [file pone.0320510.s003.tiff]

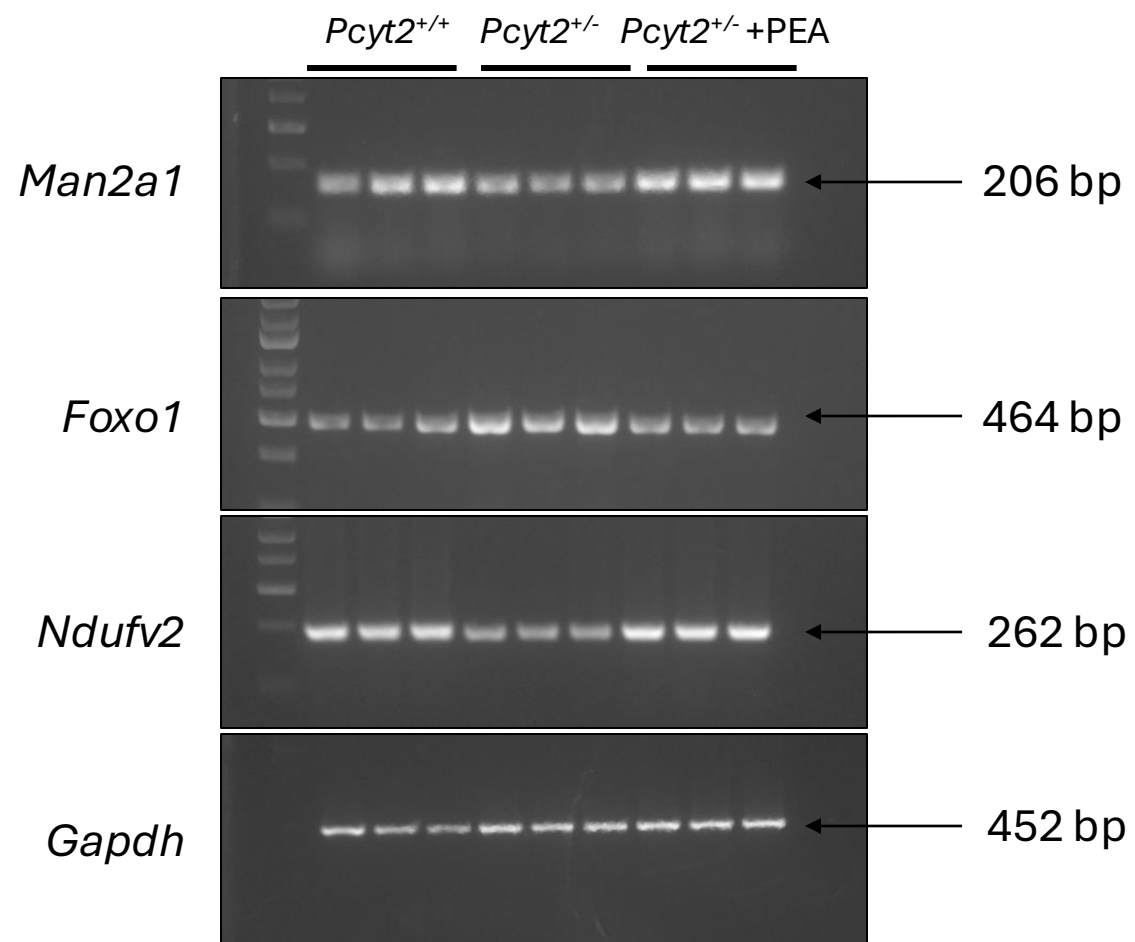

Supplement: S1 raw images — (PDF) [file pone.0320510.s005.pdf]
